# The Prevalence of Pathogens among Ticks Collected from Livestock in Kazakhstan

**DOI:** 10.3390/pathogens11101206

**Published:** 2022-10-19

**Authors:** Kulyaisan T. Sultankulova, Gaukhar O. Shynybekova, Aisha U. Issabek, Nazym N. Mukhami, Aibarys M. Melisbek, Olga V. Chervyakova, Nurlan S. Kozhabergenov, Sabyrkhan M. Barmak, Arailym K. Bopi, Zamira D. Omarova, Dana A. Alibekova, Takhmina U. Argimbayeva, Aidar M. Namet, Ivan A. Zuban, Mukhit B. Orynbayev

**Affiliations:** 1 Research Institute for Biological Safety Problems of the Ministry of Health of the Republic of Kazakhstan, Gvardeiskiy 080409, Kazakhstan; 2 LLP “Kazakh Research Institute of Processing and Food Industry”, Almaty 050060, Kazakhstan; 3 MVA Group Scientific-Research Production Center Ltd., Almaty 050046, Kazakhstan; 4 Department “Biology”, Faculty of Mathematics and Natural Sciences, M. Kozybayev North Kazakhstan University, Petropavlovsk 150000, Kazakhstan

**Keywords:** ixodid ticks, lumpy skin disease, Q fever, theileriosis, babesiosis, Kazakhstan

## Abstract

Ticks carry and transmit a wide variety of pathogens (bacteria, viruses and protozoa) that pose a threat to humans and animals worldwide. The purpose of this work was to study ticks collected in different regions of Kazakhstan for the carriage of various pathogens. The collected ticks were examined by PCR for the carriage of various pathogens. A total of 3341 tick samples parasitizing three animal species (cattle, sheep and horses) were collected at eight regions of Kazakhstan. Eight tick species were found infesting animals: *Dermacentor marginatus* (28.08%), *Hyalomma asiaticum* (21.28%), *Hyalomma anatolicum* (17.18%), *Dermacentor reticulatus* (2.01%), *Ixodes ricinus* (3.35%), *Ixodes persulcatus* (0.33%), *Hyalomma scupense* (12.87%) and *Hyalomma marginatum* (14.90%). Ticks collected from livestock animals were examined for the pathogen spectrum of transmissible infections to determine the degree of their infection. Four pathogen DNAs (lumpy skin disease virus (LSDV), *Coxiella burnetti*, *Teileria annulata*, and *Babesia caballi*) were detected by PCR in *Dermacentor marginatus*, *Hyalomma asiaticum*, *Hyalomma scupense*, *Hyalomma anatolicum*. The infection of ticks *Dermacentor marginatus* and *Hyalomma asiaticum* collected on cattle in the West Kazakhstan region with LSDV was 14.28% and 5.71%, respectively. *Coxiella burnetti* was found in the ticks *Dermacentor marginatus* (31.91%) in the Turkestan region and *Hyalomma anatolicum* (52.63%) in the Zhambyl region. *Theileria annulata* was found in ticks *Hyalomma scupense* (7.32%) and *Dermacentor marginatus* (6.10%) from cattle in the Turkestan region. *Babesia caballi* was isolated only from the species *Hyalomma scupense* (17.14%) in the Turkestan region. There were no PCR-positive tick samples collected from sheep. RNA/DNAs of tick-borne encephalitis virus (TBEV), African swine fever virus (ASFV), Hantavirus hemorrhagic fever with renal syndrome (HFRS), and chlamydia pathogens were not found in ticks. The new data give a better understanding of the epidemiology of tick-borne pathogens and the possibility of the emergence of tick-borne animal diseases in Kazakhstan.

## 1. Introduction

Ticks are significant vectors of various diseases that pose serious public health threats and significant economic losses, affecting the health and productivity of animals [1,2]. Ticks transmit a wide range of microorganisms, including protozoa, bacteria, and viruses [3].

The territory of Kazakhstan occupies from the eastern outskirts of the Volga delta in the west to the Altai Mountains in the east, from the West Siberian Plain in the north to the Tien Shan mountain system in the south of the country. The total area is 2724.9 thousand km^2^. The relief of Kazakhstan is mostly flat—more than 80% of the country is dry steppes. The diversity of landscape and climatic conditions and the animal world of the country creates the prerequisites for the existence of foci of various pathogens, primarily associated with ticks.

The tick fauna of Kazakhstan includes more than 30 species of ixodid ticks, recognized as carriers of a number of dangerous infectious pathogens. *Dermacentor marginatus*, *Hyalomma marginatum*, *Hyalomma anatolicum*, *Hyalomma asiaticum*, *Hyalomma scupense* have epidemiological significance in the territory of Kazakhstan [4]. In Kazakhstan, ticks are carriers of Crimean-Congo hemorrhagic fever (CCHF) [5], tick-borne encephalitis (TBE) [6], babesiosis and theileriosis [7], Q fever [8,9] and Lyme borreliosis [10].

Over the past few decades, an increase in both the number of ticks in Kazakhstan and the number of cases of tick-borne diseases (TBD) has been recorded [8,10,11,12,13].

Research on tick-borne diseases in Kazakhstan is rare. All research findings concerning tick-borne diseases are the results of studies performed within the framework of small scientific projects. These studies are insufficient to better understand the risk of tick-borne infections in different ecological zones. There is no state program to study the prevalence of pathogens among ticks in Kazakhstan. Therefore, many issues of the current state of tick populations and their epidemiological significance in the territory remain insufficiently studied. The current situation on the spread of ixodid ticks—potential carriers of infections—in the Republic of Kazakhstan requires further study of the ranges, abundance and infection of these arthropods, as well as the establishment of their epidemiological significance. Eliminating this deficiency is the goal of this study, studying ticks collected in different regions of Kazakhstan for the carriage of various pathogens.

## 2. Results

### 2.1. Species Composition of Ixodid Ticks in Livestock

**Figure 1:**
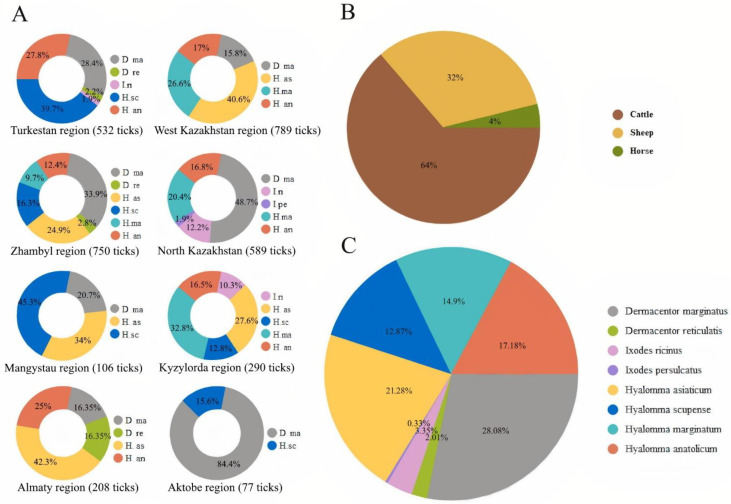
Tick species collected from animals in Kazakhstan. D. Ma—*Dermacentor marginatus*; D. re—*Dermacentor reticulatus*; I. ri—*Ixodes ricinus*; I. pe—*Ixodes persulcatus*; H. as—*Hyalomma asiaticum*; H. sc—*Hyalomma scupense*; H. ma—*Hyalomma marginatum*; H. an—*Hyalomma anatolicum*. (**A**)—species composition of ticks in regions; (**B**)—percentage of ticks found on different types of animals; (**C**)—species composition of ticks found on the territory of the country.

A total of 3341 ticks were collected from 175 cattle, 76 horses and 247 sheep. According to the study of the ixodofauna of cattle, horses and sheep in various regions of Kazakhstan in 2021 and 2022, 8 tick species of the *Ixodidae* family from 3 genera were identified: *Dermacentor* (*Dermacentor marginatus*, *Dermacentor* reticulatus), *Ixodes* (*Ixodes ricinus*, *Ixodes* *persulcatus*), *Hyalomma* (*Hyalomma marginatum*, *Hyalomma anatolicum*, *Hyalomma scupense*, *Hyalomma asiaticum*). The most abundant species found were *Dermacentor marginatus *(28.08%) and *Hyalomma siaticum *(21.28%) and *Hyalomma anatolicum* (17.18%). Other five species, i.e., *Dermacentor reticulatus* (2.01%), *Ixodes ricinus* (3.35%), *Ixodes persulcatus* (0.33%), *Hyalomma scupense* (12.87%) and *Hyalomma marginatum* (14.90%), were rarer (Figure 1).

### 2.2. PCR Detection of Tick-Borne Pathogens

The DNA of four pathogens (LSDV, *Coxiella burnetti*, *Teileria annulata* and *Babesia caballi*) was detected using PCR in *Dermacentor marginatus*, *Hyalomma asiaticum*, *Hyalomma scupense*, and *Hyalomma anatolicum* (Table 1, Figure 1).

**Figure 2:**
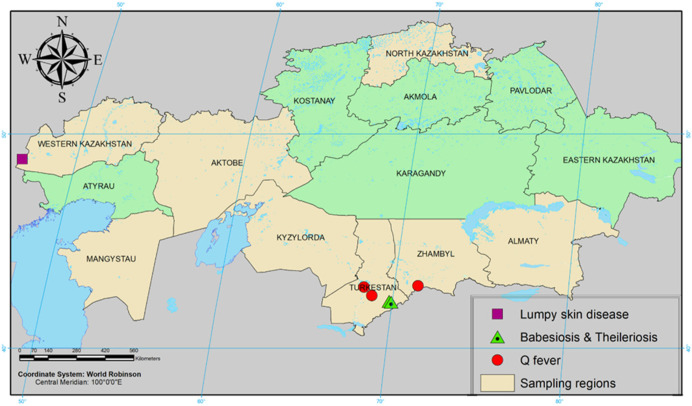
Geographical distribution of pathogens of natural focal infections in Kazakhstan, carried by ticks.

LSDV DNA was detected in *Dermacentor marginatus* (14.28%) and *Hyalomma asiaticum* (5.71%) collected from cattle in the Bokey Orda district of the West Kazakhstan region. *Coxiella burnetti* DNA was detected in *Dermacentor marginatus* (31.91%) collected from cattle in the Otrar district of Turkestan region and *Hyalomma anatolicum *(52.63%) in Taraz city of Zhambyl region. *Teileria annulata* DNA was found in *Hyalomma scupense *(7.32%) and *Dermacentor marginatus* (6.10%) collected from cattle in the Tolebi district of Turkestan region. *Babesia caballi *DNA was isolated only in *Hyalomma scupense *(17.14%) in the Tolebi district of Turkestan region (Figure 2). There were no PCR-positive tick samples collected from sheep.

RNA/DNA of TBEV, ASFV, HFRS, and chlamydia pathogens were not found in ticks.

### 2.3. Sequence Analysis of Tick-Borne Pathogens

The nucleotide sequences of *Theileria annulata *(GenBank accession number OP077209, OP077210), which encode the small ribosomal RNA subunit, are identical to strains from Pakistan (MT341858, MG599093), Turkey (MG569892), China (KU554731), India (MF287925), and Italy (MT341858).

The nucleotide sequence of *Babesia caballi *(GenBank accession number OP077204), which encodes a small ribosomal RNA subunit, is 100% identical to isolates from Israel (MN629354, MK288109) and South Africa (EU642513, MK288108).

The nucleotide sequence of LSDV (GenBank accession number OP122557, OP122558), which encodes the ankyrin-repeat protein gene, was highly identical with LSDV strain Kubash/KAZ/16 (MN642592), LSDV Kenya isolate Kenya (MN072619), LSDV strain LSDV/Russia/Dagestan/2015 (MH893760).

**Figure 3:**
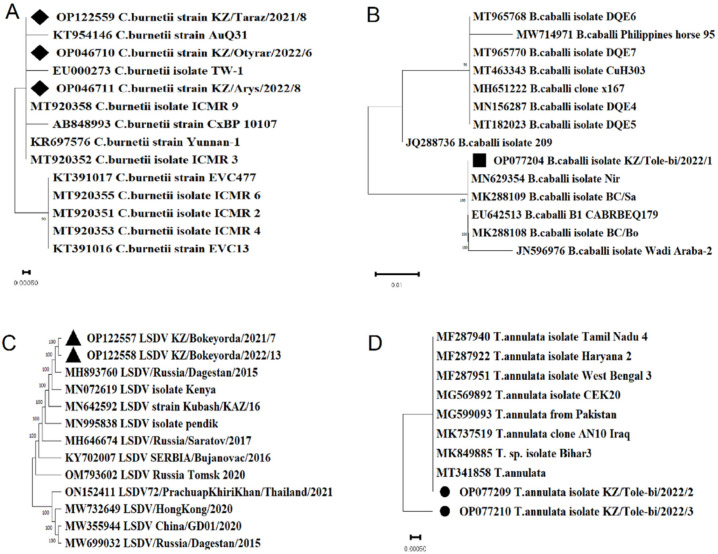
Neighbor-joining phylogenetic trees based on a fragment of the IS1111A transposase gene of *Coxiella burnetii *(**A**), a fragment of the small subunit ribosomal RNA gene of *Babesia caballi *(**B**), a fragment of the Ankyrin repeat protein gene of LSDV (**C**), a fragment of the small subunit ribosomal RNA gene of *Theileria annulata *(**D**).

As a result of data analysis of *Coxiella burnetii *(GenBank accession number OP122559, OP046711, OP046710), it was found that the sequenced region of the IS1111A gene, was highly identical with *Coxiella burnetii *strain AuQ31 (KT954146), *Coxiella burnetii *isolate TW-1 (EU000273), *Coxiella burnetii *isolate ICMR 9 (MT920358) (Figure 3).

## 3. Discussion

Ticks play a substantial role as vectors of pathogens. Pathogens transmitted by ticks are responsible for the majority of the vector-borne diseases in temperate regions of America, Europe and Asia. According to some researchers, more than 100,000 cases per year of tick-borne diseases are recorded in the world [14]. Tick-borne pathogens affect 80% of the world’s cattle and are widespread throughout the world, especially in developing countries where little attention is paid to the study of tick-borne animal diseases [15,16]. This is the first comprehensive study of tick-borne viral, bacterial, rickettsial and protozoan pathogens of human and veterinary interest in Kazakhstan.

The ticks collected in this study reinforce previously recorded distributions of tick species in Kazakhstan [4,17,18,19,20].

In the present study, 3341 ticks encompassing 8 species were collected from 8 regions of Kazakhstan: *Dermacentor marginatus* (938/28.08%), *Hyalomma asiaticum* (711/21.28%), *Hyalomma anatolicum *(574/17.18%), *Hyalomma marginatum *(498/14.90%), *Hyalomma scupense *(430/12.87%), *Ixodes ricinus *(112/3.35%), *Dermacentor reticulatus *(67/2.01%), *Ixodes persulcatus *(11/0.33%). On 175 cattle 2127 (63.66%) ticks belonging to 8 species (all of the above species), on 247 sheep 1085 (32.48%) ticks belonging to 7 tick species (all of the above species, except *Dermacentor reticulatus*) and on 76 horses 129 (3.86%) ticks belonging to 5 tick species (*Dermacentor marginatus*, *Hyalomma asiaticum*, *Hyalomma anatolicum*, *Hyalomma marginatum*, *Hyalomma scupense*) were identified.

In our study, four species of ticks (*Dermacentor marginatus*, *Hyalomma asiaticum*, *Hyalomma anatolicum*, *Hyalomma scupense*) out of eight tested were PCR-positive and considered as vectors of pathogens among domestic animals. Lumpy skin disease, Q fever, babesiosis, and theileriosis viral DNAs were detected in ticks from various regions of Kazakhstan.

Lumpy skin disease (LSD) is an infectious disease that is transmitted by various arthropods [21]. Almost to the end of the last century, the disease was previously reported only in the countries of Central and South Africa, and only in the 1980s was it first introduced to the Middle East [22]. In 2014, the disease spread to Azerbaijan [23], then from there, it spread to Russia and resulted in significant economic losses [24]. In the European Union, namely Greece, LSD was discovered in 2015. In 2016, the list had grown, with Armenia, Bulgaria, Serbia, Albania and Kazakhstan [24]. Later, LSD outbreaks were reported in Bangladesh, India, China, Nepal, Bhutan, Vietnam, Myanmar, Sri Lanka, Thailand, Malaysia, and Laos [25,26].

Until 2015, the disease had not been reported in northern temperate regions and there were many gaps in the knowledge about potential carriers of LSDV. The first report on the transmission of LSDV by ticks in northern latitudes was confirmed after LSD outbreaks in Russia. Viral DNA has been detected at least in 13 species of ixodid ticks belonging to six genera. LSDV genome has been detected in *Ixodes ricinus *(16.3% of the total studied ticks), Boophilus annulatus (14.3%), *Dermacentor marginatus* (13.8%), *Hyalomma marginatum *(11.6%), and Haemaphysalis scupense (8.1%). This led to the conclusion that ixodid ticks may have acted as LSDV reservoirs during the 2015 outbreaks, but more detailed studies are required to confirm these preliminary results [27]. In our studies, LSDV DNA was detected from the tick species *Dermacentor marginatus* (14.28%) and *Hyalomma asiaticum* (5.71%) collected from cattle in the West Kazakhstan region. These data are consistent with the results of our previous studies, where it was shown that *Dermacentor marginatus* and *Hyalomma asiaticum* ticks taken from sick animals during the LSD epizootics in the Atyrau region were involved in LSDV transmission [28]. The presence of PCR-positive ticks in the western regions of Kazakhstan is possibly associated with the movement of wild animals, which are possibly asymptomatic carriers of this disease. We hypothesize that ticks remained infected after feeding with infected blood during the outbreak in Atyrau region of Kazakhstan or the border regions of Russia. Subsequently, they spread to the territory of the West Kazakhstan region through wild animals. Currently, the largest population of saigas (about 1.0 million individuals) inhabits Western Kazakhstan, which migrates through the territory of Atyrau and West Kazakhstan regions, as well as the border regions of the Russian Federation and possibly may transmit the LSDV from infected ticks. To confirm this hypothesis, it is necessary to conduct monitoring studies both in the saiga population and in other wild artiodactyl animals.

Q fever is an infectious disease caused by *Coxiella burnetii *that is prevalent throughout the world and has a significant impact on animal welfare and human health [29,30]. The outbreak of Q fever in the Netherlands is an example of this [31]. Between 2007 and 2010, during the Q fever epidemic in the Netherlands, more than 4000 human cases were confirmed and over 50,000 dairy goats were slaughtered. From 2011 to 2016, Germany had the highest prevalence of Q fever, with an average of 240 cases per year (incidence of 2 per 100,000), followed by France, Spain and Hungary, with 180, 110 and 60 cases per year, respectively [32]. In China, there have been 29 reports of Q fever over the last 25 years (1989–2013), almost half of which occurred over the last 5 years [33]. The overall prevalence of *Coxiella burnetii *infection in China is 10% in humans, 15% in cattle, and 12% in goats.

However, knowledge of *Coxiella burnetii *remains limited to this day. Besides the main route of transmission via inhalation of infectious aerosols, ticks are still under debate as potential vectors for *Coxiella burnetii*. The importance of ticks in the epidemiology of Q fever remains controversial, although a sufficient number of studies have shown the presence of *Coxiella burnetii *in ticks [34]. Recent studies have shown the prevalence of 4.8% of *Coxiella burnetii *infection among 25 different tick species in 23 European countries. Significantly higher prevalence was observed in southern European countries [34].

In China, only one study detected *Coxiella burnetii *specific DNA in eight tick species (*Dermacentor silvarum*, *Dermacentor niveus*, *Dermacentor nuttalli*, *Hyalomma detritum*, *Hyalomma scupense*, *Haemaphysalis japonica*, *Hyalomma concinna*, and *Hyalomma qinghaiensis*) from four provinces in Northwest China. Recent studies have shown that the prevalence of *Coxiella burnetii *in ticks in four provinces in Northwest China averages 10%, 11.9% in Xinjiang and 2% in three northeastern provinces (Jilin, Liaoning, Heilongjiang) and Inner Mongolia. *Coxiella burnetii *DNA was found in *Dermacentor silvarum*, *Ixodes persulcatus*, Hyalomma conicinna, Hyalomma japonica, Boophilus microplus, *Dermacentor nuttalli*, *Dermacentor niveus*, *Hyalomma detritum*, *Hyalomma scupense*, and *Hyalomma qinghaiensis* [33].

Q fever has been reported in Kazakhstan since the early 1950s [35]. There are no data about the prevalence of Q fever among wild and domestic animals in the territory of Kazakhstan in the available literature. There are separate records on the identified seropositivity for *Coxiella burnetii *in saigas [36]. There is very little research evidence of ticks for the carriage of *Coxiella burnetii *in various regions of the country. Our studies showed PCR-positive results for *Coxiella burnetii *in *Dermacentor marginatus* (31.91%) collected in Turkestan region and *Hyalomma anatolicum *(52.63%) in the Zhambyl region. These data are consistent with the results of our previous studies [9].

The authors have shown that in 2013, in the Kyzylorda region, the tick infestation of *Coxiella burnetii *in *Hyalomma asiaticum* and *Dermacentor marginatus* was 4% and 2.3%, respectively. In the present study, we did not detect positive samples in other regions of Kazakhstan. However, according to some researchers, *Coxiella burnetii *was previously detected in the northern and western regions [37]. To obtain a comprehensive assessment of the prevalence of *Coxiella burnetii *in various regions of Kazakhstan, it is necessary to continue monitoring studies with a larger number of samples.

Theileriosis is distributed throughout the world, from Asia, the Middle East and South Europe to North Africa [38,39] and is transmitted by several types of hard (Ixodidae) ticks: *Hyalomma anatolicum*, Hyalomma lusitanicum, *Hyalomma scupense*, *Hyalomma detritum detritum*, and *Hyalomma dromedarii* [40,41,42]. According to some researchers, the prevalence of theileriosis in cattle in China was 39%, Iran 33%, India 31.7%, Pakistan 21.2%, Bangladesh 2.69% [42], and Egypt 16.49% [43].

The prevalence of diseases caused by blood parasites in Kazakhstan is poorly understood. There are isolated reports indicating the presence of these diseases among animals and ticks in Kazakhstan [7,44].

Studies conducted by a group of scientists from Kazakhstan, China and Hungary showed that *Theileria annulata *are present among ticks Rhipicephalus turanicus in the Almaty region, *Dermacentor marginatus* in the Turkestan region and Rhipicephalus turanicus in the Zhambyl region. The prevalence among ticks ranged from 2.30% to 33.33% [8]. In our studies, *Theileria annulata *DNA was detected in *Hyalomma scupense *(7.32%) and *Dermacentor marginatus* (6.10%) tick species collected from cattle. All PCR-positive ticks were collected in Turkestan region. These results are consistent with the results of our previous studies [7] and confirm that *Dermacentor marginatus* is the main carrier of *Theileria annulata *in Turkestan region. The detection of theileria in *Hyalomma scupense *indicates that this tick species is a potential vector for this disease.

Equine Piroplasmosis (EP) is widespread in almost all countries of the world and cause enormous harm to agriculture [45,46]. Animals of each species have their own specific pathogens. The causative agents of equine piroplasmosis are *Babesia caballi *and *Theileria equi*. Equine babesiosis is endemic in most countries of the tropical and subtropical regions of the world [47,48,49]. However, there is reported evidence of the presence of this disease in EU countries, such as the Netherlands and Italy [50,51]. The global spread and distribution of equine piroplasmidoses depends on the availability of competent tick vectors capable of transmitting these pathogens to horses. Ticks are the definitive hosts and vectors of *Babesia caballi *and *Theileria equi*. Scoles and Ueti described 33 Ixodid tick species belonging to six genera as competent vectors responsible for equine piroplasmosis [52]. The authors have shown that ticks belonging to the genera *Hyalomma*, *Dermacentor*, *Rhipicephalus* are the biological vectors of *Theileria equi* and *Babesia caballi*, while other genera of ticks, including *Amblyomma*, *Haemaphysalis* and *Ixodes*, are suspected but not confirmed. The list is probably not exhaustive since most research studies are focused on only a small subset of ticks closely related to horses.

The presence of *Theileria* species in livestock and wild animals or their ticks is not well studied in our country. Recent studies have suggested that causative agents of piroplasmosis in horses are present in ticks in the territory of Almaty and Turkestan regions [7]. In their study, Sang C. et al. showed the presence of *Babesia caballi *DNA in one *Dermacentor marginatus* tick and *Theileria equi* DNA in two *Hyalomma asiaticum* ticks in the Almaty region. The same study also revealed that *Babesia caballi *DNA was found in 2 ticks and *Theileria equi* DNA in 6 *Dermacentor marginatus* ticks in Turkestan region.

In our studies, the DNA of *Babesia caballi *was isolated only from the *Hyalomma scupense *(17.14%) specie collected from horses. All PCR-positive ticks were collected in Turkestan region. Based on the data obtained, it can be assumed that *Hyalomma scupense *is one of the likely vectors of this disease. To confirm these data, it is necessary to continue studies on a larger number of samples.

Our results have increased awareness about the distribution of tick-borne pathogens in Kazakhstan. However, study results on tick distribution, population and disease presence are insufficient in Kazakhstan to better understand the risk of tick-borne infections within various ecologic zones. Further research on ticks in different regions is needed to assess the current situation in the country. Further research on ticks in different regions is required to assess the current situation in the country.

## 4. Materials and Methods

### 4.1. Study Area and Collection of Samples

We conducted a field sampling of ticks in April, May of 2021 and 2022 in rural area in Turkestan, West Kazakhstan, Zhambyl, North Kazakhstan and Almaty regions. A total of 3341 ixodid ticks (*Dermacentor marginatus*, *Dermacentor reticulatus*, *Ixodes ricinus*, *Ixodes persulcatus*, *Hyalomma asiaticum*, *Hyalomma scupense*, *Hyalomma marginatum*, and *Hyalomma anatolicum*) were collected from animals (Table 2).

Adult ticks were sampled using tweezers directly from cattle, horses, and sheep of both sexes. Ticks were collected from different body parts of animals as the inner side of the thigh, udder, scrotum, neck, and armpit of the animals. Ticks were transported for analysis on the day of collection. In the absence of such a possibility, adult ticks were maintained alive in plastic vials with grass for 10 days in a cool place or refrigerated. Each vial contained a detailed label with the following information for each included sample: date, type and number of examined animals, and the collection place. Each arthropod was identified using the stereomicroscope RS0745 (Altami, Saint Petersburg, Russia). Species identification of ticks was confirmed morphologically [53,54].

While sampling and accounting, sampling staff had to follow special precautions as wearing a personal protective clothing with a high neckline and cuffs, also periodic self- and mutual inspections for the presence of ticks.

### 4.2. Sample Preparation and DNA/RNA Purification

After morphological evaluation, ticks were disinfected with 70% ethanol, then rinsed in ultrapure water, again treated with 70% ethanol, and homogenised in PBS [55]. The resulting tick suspension was stored at −80 °C until testing.

Each tick was processed separately, according to the principle “one tick—one sample”. DNA/RNA was isolated from each tick.

Extraction of nucleic acids from tick suspensions (*Dermacentor marginatus*, *Dermacentor reticulatus*, *Ixodes ricinus*, Ixodes persulcatus, *Hyalomma asiaticum*, *Hyalomma scupense*, *Hyalomma marginatum*, *Hyalomma anatolicum*) was carried out using commercial TRizol reagent (Invitrogen, Carlsbad, CA, USA) in accordance with manufacturer’s instructions. DNA/RNA concentrations were measured using a NanoDrop 2000 spectrophotometer (Thermo Fisher Scientific, Waltham, MA, USA). To assess the purity and quality of nucleic acids in spectrophotometric measurement, the purity of the sample was determined based on the ratio of optical densities at A260/A280.

### 4.3. Confirmation of the Presence of Tick-Borne Pathogens by PCR Method

Classical PCR was performed in a GeneAmp PCR System 9700 thermal cycler (Applied Biosystems, Foster City, CA, USA). RT-PCR was performed using a Rotor-Gene Q thermal cycler (Qiagen, Hilden, Germany).

*PCR to detect theileria*. Theileria species verification was performed using the AccuPrime™ Taq DNA Polymerase System PCR kit (Invitrogen, Carlsbad, CA, USA). Species-specific primers targeting the 18S rRNA gene fragment ThEq F1 (AGACAGAGG AAG GAT TGACA) and ThEqR (CTGATGACTTGCGCATACTA), which can amplify a product of 386 bp, were used. Plasmid DNA containing inserts corresponding to the *Theileria* spp. 18S rRNA gene was used as a positive control.

Each PCR reaction was carried out in a 25 μL reaction volume, which contained 2.5 µL 10× PCR buffer, 1 µL of MgCl_2_ (50 mM), 1 µL of 20 pmol F primer, 1 µL of 20 pmol R primer, 0.5 µL of Taq DNA Polymerase, 16 µL of deionized water, 3 µL of DNA. Reactions were performed under the following conditions: initial denaturation 94 °C for 2 min, followed by 35 cycles of denaturation 94 °C for 20 s, annealing at 55 °C for 30 s, extension at 68 °C for 60 s, followed by a final extension at 68 °C for 7 min.

*PCR to detect babesia*. PCR analysis was performed in a final volume of 25 µL which contained 2.5 µL of 10× PCR buffer, 1 µL of MgCl_2_ (50 mM), 1 µL of 20 pmol F primer, 1 µL of 20 pmol R primer, 0.5 µL of Taq DNA Polymerase, 16 µL of deionized water, 3 µL of DNA. AccuPrime™ Taq DNA Polymerase System PCR Kit (Invitrogen, Carlsbad, CA, USA). Detection of *Babesia caballi *was carried out using the primers B.Cab_F (TCAGCACCTTGAGAGAAATC) and B.Cab_R (ACAGATTACCCACACCTTTC), which amplify a product of 451 bp. Plasmid DNA containing inserts corresponding to the gene for the small subunit of ribosomal RNA of *Babesia* spp. was used as a positive control. Amplification conditions were as follows: initial denaturation at 94 °C for 3 min, followed by 35 cycles of denaturation at 94 °C for 20 s, annealing at 55 °C for 30 s, extension at 72 °C for 40 s, and then a final extension at 72 °C for 7 min.

*PCR to detect the LSDV*. The reaction mixture consists of 25 µL total volume: 2.5 µL of 10× PCR buffer, 1 µL of dNTP (10 mM), 2 µL of MgCl_2_ (25 mM), 1 µL of 20 pmol F primer, 1 µL of 20 pmol R primer, 0.5 µL of 5 units Taq DNA Polymerase, 14 µL of deionized water, 3 µL of DNA. The AccuPrime™ Taq DNA Polymerase System kit (Invitrogen, Carlsbad, CA, USA) was used to formulate the PCR mixture. Thermal cycling conditions were as follows: 3 min at 94 °C 35 cycle, 20 s at 94 °C, 30 s at 55 °C, 40 s at 72 °C and 7 min at 72 °C. Primers LSDV F-CTGCAAAGGCGGATAATTATGATG and LSDV R- CCATGGTGTCTTATGACCCCAAT were used for PCR. The size of the PCR product is 742 bp. When setting up PCR, the DNA of the Dermatitis nodulares/2016/Atyrau/KZ LSDV strain was used as a positive control.

*PCR to detect Coxiella burnetti*. The amplification was performed in a total volume of 25 µL, containing 3 µL of DNA sample, 2.5 µL of 10× Buffer, 1 µL of 50 mM MgCl_2_, 1 µL of primer Trans1, 1 µL of primer Trans2 and 0.5 µL of Taq DNA polymerase (AccuPrime ™ Taq DNA Polymerase System Invitrogen, Carlsbad, CA, USA). The Trans-PCR thermal program using primers Trans 1: 5′-TGG TAT TCT TGC CGA TGA C-3′; Trans 2: 5′-GAT CGT AAC TGC TTA ATA AAC CG-3′ was carried out under the following conditions: initial denaturation at 95 °C for 5 min followed by 40 cycles consisting of denaturation at 95 °C for 30 s, annealing at 60 °C for 30 s and extension at 72 °C for 1 min, then a final extension at 72 °C for 7 min [56]. When setting up PCR, the DNA of strain VR-615 16S, *Coxiella burnetii *was used as a positive control.

*PCR to detect chlamydia*. The reaction was performed in a final volume of 25 µL containing 2.5 µL of 10× PCR buffer, 1 µL of MgCl_2_ (50 mM), 1 µL of 20 pmol F primer, 1 µL of 20 pmol R primer, 0.5 µL of Taq DNA Polymerase, 16 µL of deionized water, 3 µL of DNA. AccuPrime™ Taq DNA Polymerase System kit (Invitrogen, Carlsbad, CA, USA) and primers hi1f (GCA GTC GAG AAT CTT TCG CAA TG), hi1r (AGC TGC TGG CAC GGA GTT AG) were used to compile the PCR mixture. When setting up PCR, the DNA of *Chlamydia trachomatis* serovar L2 strain 434 was used as a positive control.

The thermal profile comprised 2 min at 94 °C, followed by 35 cycles of 30 s at 94 °C, 45 s at 58 °C, 60 s at 68 °C, and 7 min at 68 °C.

*PCR to detect TBEV*. TBEV-RNA was detected by a set of reagents “OM-Screen-TBE-RT” (Syntol, Moscow, Russia). RT-PCR was performed by sequentially adding 15 µL of the diluent to the reaction mixture in stranded microtubes, 20 µL of negative control, 20 µL of samples in the required repetition, and then 20 µL of TBE positive control. PCR amplification program: 50 °C for 15 min, 95 °C for 5 min, 50 cycles (60 °C for 40 s, 95 °C for 15 s), fluorescence signal reading at 60 °C.

*PCR for the detection of ASFV*. PCR was performed using the African swine fever (ASF) test system for the detection of African swine fever virus by polymerase chain reaction (AmpliSens, Moscow, Russia). The reaction mixture consists of 25 µL total volume: 3.5 µL of PCR-c-1 ASFV, 10 µL of 2.5× PCR blue buffer, 0.5 µL of TaqF polymerase, 1 µL of UdG-TL enzyme, 10 µL of DNA, 10 µL of NC- negative control, 10 µL of ASFV DNA FRP positive control. PCR amplification program: 1 min at 95 °C, followed by 40 cycles of 10 s at 95 °C, 25 s at 62 °C, 25 s at 72 °C and 1 min at 72 °C.

*PCR for the detection of HFRS*. For the detection of HFRS, a set of reagents “OM-Screen-HFRS-RT” (Syntol, Moscow, Russia) was used. Real-time PCR is performed by serially adding 15 µL of diluent (RB) to the reaction mixture in stranded microtubes (RM-HFRS), then adding 20 µL of negative control, 20 µL of test samples, and then 20 µL of positive control. PCR amplification program: 1st stage: 50 °C, 15 min; 2nd stage: 95 °C–5 min; 3rd stage: 50 cycles (58 °C–20 s, 94 °C–15 s, 72 °C–20 s), fluorescence signal reading at 58 °C.

### 4.4. Sequencing Assays, BLASTn Analysis and Phylogenetic Analyses

For sequencing, obtained PCR products were used as the template. Sequencing was performed on an Applied Biosystems 3130 automated DNA sequencer (Hitachi, Tokyo, Japan) using the BigDye Terminator v3.1 Cycle Sequencing kit (Applied Biosystems, Inc., Vilnius, Lithuania). The resulting nucleotide sequences were analyzed in Sequencer v. 4.5 (Gene Codes Corporation, Ann Arbor, MI, USA).

The identities and similarities of sequenced isolates were analyzed using the basic local alignment search tool (BLASTn) of the National Center for Biotechnology Information (NCBI) GenBank database and MEGA 11.0 program [57].

The analysis used available nucleotide sequences of strains in GenBank: *Theileria annulata *(MF287940, MF287922, MF287951, MG569892, MG599093, MK737519, MK849885, MT341858), *Babesia caballi *(MT965768, MW714971, MT965770, MT463343, MH651222, MN156287, MT182023, JQ288736, OP077204, MN629354, MK288109, EU642513, MK288108, JN596976, LSDV (MH893760, MN072619, MN642592, MN995838, MH646674, KY702007, OM793602, ON152411, MW732649, MW355944, MW699032), *Coxiella burnetii *(KT954146, EU000273, MT920358, AB848993, KR697576, MT920352, KT391017, MT920355, MT920351, MT920353, KT391016).

## Figures and Tables

**Table 1:** PCR detection of pathogens in ticks depending on locality and tick species.

Scheme				Positive Ticks per Pathogen Species (%)			
Tick	Location	Host	No.	LSDV	*Coxiella burnetti*	*Teileria annulata*	*Babesia caballi*
D. ma	Bokey Orda district, West Kazakhstan region	Cattle	49	7 (14.28)	-	-	-
H. as	Bokey Orda district, West Kazakhstan region	Cattle	105	6 (5.71)	-	-	-
D. ma	Otrar district, Turkestan region	Cattle	47	-	15 (31.91)	-	-
H. sc	Tolebi district, Turkestan region	Horse	35	-	-	-	6 (17.14)
H. sc	Tolebi district, Turkestan region	Cattle	82	-	-	6 (7.32)	-
D. ma	Tolebi district, Turkestan region	Cattle	64	-	-	5 (6.10)	-
H. an	Taraz city, Zhambyl region	Cattle	19	-	10 (52.63)	-	-

Note: “-” means not detected; D. ma—*Dermacentor marginatus*; H. as—*Hyalomma asiaticum*; H. sc—*Hyalomma scupense*; H. an—*Hyalomma anatolicum*.

**Table 2:** List of tick species and animals from which they were collected in Kazakhstan.

Host			Tick Species							
Location	Host Animals	No.	D. ma	D. re	I. ri	I. pe	H. as	H. sc	H. ma	H. an
Otrar district, Turkestan region N 42°51′25″ E 68°3′5″	Cattle	13	47	12	10	-	-	42	-	72
	Sheep	15	13	-	-	-	-	25	-	34
	Horse	6	7	-	-	-	-	-	-	3
Tolebi district, Turkestan region N 42°10′59″ E 69°52′57″	Cattle	15	64	-	-	-	-	82	-	32
	Sheep	16	12	-	-	-	-	27	-	7
	Horse	5	8	-	-	-	-	35	-	-
Bokey Orda district, West Kazakhstan region N 48°57′22″ E 47°36′46″	Cattle	14	49	-	-	-	105	-	75	28
	Sheep	20	23	-	-	-	56	-	27	46
	Horse	6	10	-	-	-	14	-	-	3
Zhanibek district, West Kazakhstan region N 49°27’00″ E 46°53’24″	Cattle	15	22	-	-	-	89	-	83	32
	Sheep	20	21	-	-	-	47	-	25	25
	Horse	6	-	-	-	-	9	-	-	-
Korday district, Zhambyl region N 43°2′42″ E 74°42′27″	Cattle	12	92	21	-	-	38	34	12	37
	Sheep	16	34	-	-	-	35	31	-	25
	Horse	5	4	-	-	-	12	-	2	-
Taraz city, Zhambyl region N 42°52′48″ E 71°21′47″	Cattle	24	78	-	-	-	67	22	34	19
	Sheep	17	46	-	-	-	35	30	23	12
	Horse	6	-	-	-	-	-	5	2	-
Timiryazev district, North Kazakhstan region N 53°48′00″ E 66°32′24″	Cattle	12	103	-	40	4	-	-	30	27
	Sheep	33	52	-	17	-	-	-	25	19
	Horse	7	-	-	-	-	-	-	-	-
Taiynsha district, North Kazakhstan region N 53°52′48″ E 69°43′48″	Cattle	10	95	-	15	7	-	-	40	35
	Sheep	22	37	-	-	-	-	-	25	18
	Horse	5	-	-	-	-	-	-	-	-
Zhambyl district, Almaty region N 43°16′17″ E 76°40′15″	Cattle	15	34	28	-	-	56	-	-	28
	Sheep	25	-	6	-	-	32	-	-	15
	Horse	6	-	-	-	-	-	-	-	9
Zhalagash district, Kyzylorda region N 45°4′49″ E 64°40′43″	Cattle	10	-	-	30		34	15	50	25
	Sheep	16	-	-	-		46	22	45	23
	Horse	5	-	-	-		-		-	-
Munaily district, Mangystau region N 43°41′44″ E 51°19′34”	Cattle	15	10	-	-	-	15	23	-	-
	Sheep	18	-	-	-	-	-	11	-	-
	Horse	6	-	-	-	-	-	-	-	-
Mangystau district, Mangystau region N 43°41′44″ E 51°19′34″	Cattle	10	12	-	-	-	13	14	-	-
	Sheep	14	-	-	-	-	8	-	-	-
	Horse	7	-	-	-	-	-	-	-	-
Mugalzhar district, Aktobe region N 48°35′9″ E 58°27′44″	Cattle	10	34	-	-	-	-	12	-	-
	Sheep	15	25	-	-	-	-	-	-	-
	Horse	6	6	-	-	-	-	-	-	-
			938 (28.08%)	67 (2.01%)	112 (3.35%)	11 (0.33%)	711 (21.28%)	430 (12.87%)	498 (14.90%)	574 (17.18%)

Note: “-” mean not detected; D. ma—*Dermacentor marginatus*; D. re—*Dermacentor reticulatus*; I. ri—*Ixodes ricinus*; I. pe—*Ixodes persulcatus*; H. as—*Hyalomma asiaticum*; H. sc—*Hyalomma scupense*; H. ma—*Hyalomma marginatum*; H. an—*Hyalomma anatolicum*.

## Data Availability

Not applicable.
